# Efficacy of frovatriptan as compared to other triptans in migraine with aura

**DOI:** 10.1186/s10194-015-0514-8

**Published:** 2015-04-01

**Authors:** Stefan Evers, Lidia Savi, Stefano Omboni, Carlo Lisotto, Giorgio Zanchin, Lorenzo Pinessi

**Affiliations:** Department of Neurology, University of Münster, Münster, Germany; Department of Neurology, Krankenhaus Lindenbrunn, Coppenbrügge, Germany; Department of Neurology, University of Turin, Turin, Italy; Italian Institute of Telemedicine, Varese, Italy; Department of Neurology, Hospital of Pordenone, Pordenone, Italy; Department of Neurology, University of Padova, Padova, Italy

**Keywords:** Frovatriptan, Almotriptan, Zolmitriptan, Rizatriptan, Migraine with aura, Metaanalysis

## Abstract

**Background:**

The treatment of migraine attacks with aura by triptans is difficult since triptans most probably are not efficacious when taken during the aura phase. Moreover, there are insufficient data from randomised studies whether triptans are efficacious in migraine attacks with aura when taken during the headache phase. In this metaanalysis, we aimed to compare the efficacy of frovatriptan versus rizatriptan, zolmitriptan, and almotriptan.

**Methods:**

Five double-blind, randomized, controlled crossover trials were pooled. All trials had an identical design. Patients were asked to treat three consecutive migraine attacks with frovatriptan 2.5 mg and three consecutive migraine attacks with a comparative triptan (rizatriptan 10 mg; zomitriptan 2.5 mg; almotriptan 12.5 mg).

**Results:**

In this analysis, 117 migraine attacks with aura could be included (intention-to-treat population). The mean headache intensity after 2 hours was 1.2 +/- 1.0 for frovatriptan and 1.6 +/- 1.0 for the other triptans (p<0.05); all triptans showed significant improvement of headache. Frovatriptan resulted in significantly lower relapse rates at 24 hours and 48 hours when taken in migraine attacks with aura.

**Conclusions:**

Our data suggest that frovatriptan is efficacious and even superior in some endpoints also when taken during the headache phase in migraine attacks with aura. This is of particular importance for those many patients who have migraine attacks both without and with aura.

## Background

The efficacy of triptans in migraine with aura refers to different questions. First, it is of interest whether triptans are able to treat the aura symptoms [1]. Second, it has been studied whether triptans taken during the aura phase of a migraine attack are efficacious to treat the headache [1-4], which is not recommended in treatment guidelines [5]. Further, triptans are not approved to be taken during the aura phase because of their vasoconstrictive properties. Third, it is of interest whether triptans are efficacious against the headache in migraine attacks both without and with aura when taken in the headache phase. Since many patients have both types of attacks, this refers to reliability of triptan efficacy. Beside pain-free and abrupt relief from pain, this is a very important parameter for patients [6-8]. This is also expressed in another study, when 55% of the patients would prefer a long-acting triptan versus a rapid-onset, short-acting agent [9]. The very recent guideline of the International Headache Society (IHS) for controlled trials of drugs in migraine defined consistency as one of the secondary parameters for the evaluation of results [10].

Frovatriptan is a potent 5-HT1_B//D_ receptor agonist and has the highest 5-HT1_B_ potency in the triptan class; preclinical pharmacodynamic studies demonstrated that frovatriptan is apparently cerebroselective [11]. In clinical pharmacology studies, frovatriptan was shown to have a long terminal elimination half-life time of 26 hours [11,12]. This could be an argument for better clinical consistency. However, a direct comparison of frovatriptan to different other triptans with respect to efficacy in migraine attacks with aura is still missing.

Since frovatriptan has shown advantages in some outcome parameters in a large study program comparing frovatriptan to other triptans [13], we were interested in whether this is also true when treating migraine attacks with aura. Therefore, we performed a metaanalysis of all those trials with a head-to-head comparison of frovatriptan to another triptan in the acute treatment of migraine attacks with aura. The aim of the study was to compare the efficacy of the different triptans in the treatment of these specific attacks with respect to headache. This analysis did not aim to evaluate the efficacy of triptans when taken during the aura phase or the efficacy of triptans against the aura symptoms.

## Methods

This study is based on five trials which compared frovatriptan to rizatriptan (two trials), zolmitriptan (two trials), and almotriptan (one trial), respectively. All these trials were double-blind, randomized crossover trials. Three were Italian trials and already published [14-16]. Two were European trials not yet published as a full paper (complete data on file). All trials were approved by the local ethics committees. All patients gave written informed consent before randomization.

The trial design of these five trials was nearly identical and described previously [14-16]. In brief, patients aged ≥18 and ≤65 years with a current history of migraine with or without aura according to the IHS criteria [17] and having experienced an average of at least one but not more than six migraine attacks per month for six months prior to entry into the study were enrolled. Exclusion criteria were a history suggestive of ischaemic heart disease or any atherosclerotic disease indicating an increased risk of coronary ischaemia; symptomatic cardiac arrhythmias; history of stroke or transient ischaemic attack (TIA); uncontrolled hypertension; history of basilar, hemiplegic, or ophthalmoplegic migraine; severe liver and renal impairment; renal disease, or renal failure; known or suspected intolerance of, or hypersensitivity, or contraindications to any component of the trial medications; use of either test medication to treat any one of the last three episodes of migraine; history of intolerance or inefficacy of at least two triptans for the treatment of migraine attacks; abuse of alcohol, analgesics or psychotropic drugs; any severe concurrent medical condition that, according to the site investigator, may affect the interpretation of clinical trial results; pregnancy or breastfeeding; inability or unwillingness to issue the informed consent; more than six days per month of tension-type headache.

Patients complying with these inclusion/exclusion criteria were randomised 1 to 1 within each centre with a predetermined randomisation list in balanced blocks, to receive frovatriptan 2.5 mg or rizatriptan 10 mg, zolmitriptan 2.5 mg, and almotriptan 12.5 mg, respectively. Prior to randomisation the patients were monitored for migraine history including the MIDAS questionnaire, medical history, medications history, vital signs. If applicable, a pregnancy test was performed.

The assigned treatment was to be taken in three consecutive attacks of migraine. A patient could use up to two doses two hours apart to treat an attack, and up to two doses every 24 hours for episodes lasting more than one day. The three episodes should occur in a period not exceeding three months after randomisation. During each episode, the patient recorded on a diary the intensity of migraine pain from immediately before taking the medication up to 48 hours. The patient also recorded the use of medication, the possible relapse including time of relapse, and any possible adverse event.

After having treated three episodes, the patient switched to the alternative treatment, respectively, the other triptan or frovatriptan 2.5 mg. On this occasion, adverse events were reviewed, medication history checked and vital signs monitored. The patient treated the subsequent three consecutive attacks of migraine with the treatment received for the second period, with the same provisions as above regarding the dosing. The three episodes should also occur in a period not exceeding three months after switchover. After having treated three episodes with the second medication, the patient concluded the study. On this occasion, adverse events were reviewed, medication history checked, and vital signs monitored.

In this post-hoc analysis, we included all attacks in which an aura preceded the onset of the migraine headache (i.e. before the intake of the study drug). Patients were advised to take the study drug only when the migraine headache was beginning and not during the aura. However, it could be possible that the aura was still ongoing when the study drug was taken.

We evaluated the efficacy rate of the study drug for pain free at 2/4/24/48 hours after drug intake as primary endpoint; further we evaluated the mean headache intensity according to a grading from 0 to 3 (0 = none; 1 = mild; 2 = moderate; 3 = severe) and the 24 hour and 48 hour relapse rate. Statistical comparison among the treatments was made between the combined results from all five trials. Secondary endpoint was the mean headache intensity at different time points which was analysed by ANOVA. Percentages were compared using Chi^2^-test. Significance level was set at p = 0.05.

## Results

The baseline characteristics including the MIDAS score [18] of all study participants (intention-to-treat population) who treated at least one migraine attack with aura are presented in Table 1. The data are pooled according to the comparative triptan. There were no significant differences in these demographic data between the five trials analysed in this study. In total, 117 migraine attacks with aura were included into this analysis (frovatriptan = 57; rizatriptan = 28; zolmitriptan = 24; almotriptan = 8).

**Table 1:** **Baseline characteristics of the patients included in this analysis (i.e., all patients experiencing an aura before at least one attack treated with study drug) presented separately for the four different triptans**

		**Rizatriptan**	**Zolmitriptan**	**Almotriptan**	**Frovatriptan**
		**(n = 28)**	**(n = 24)**	**(n = 8)**	**(n = 57)**
Age (years)		43 +/− 9	35 +/− 10	37 +/− 11	41 +/− 11
Females		89%	88%	100%	91%
MIDAS	grade I	4%	0%	0%	4%
	grade II	4%	0%	0%	6%
	grade III	36%	55%	38%	43%
	grade IV	57%	46%	63%	48%
Attack duration >2 days		46%	41%	63%	44%

Data are shown as mean (+/− SD), or frequency in %. There were no significant differences.

Headache intensity when taking the study drug was not significantly different between the four triptan treatments (Table 2). The 2 hour and 4 hour pain free rate and the relapse rate for 24 hours and 48 hours are presented in Table 2. After 2 hours, more attacks were pain free after frovatriptan as compared to rizatriptan. There was a significantly lower percentage of relapse in attacks with aura treated with frovatriptan than in attacks with aura treated with the other triptans, both for the 24 and 48 hours endpoint (except for the comparison with almotriptan at 48 hours).

**Table 2:** **Pain free rate at 2 hours and headache recurrence rate at 24 hours and 48 hours for all migraine with aura attacks**

		**Rizatriptan**	**zolmitriptan**	**Almotriptan**	**Frovatriptan**	**Significance**
		**(n = 28)**	**(n = 24)**	**(n = 8)**	**(n = 57)**	
Baseline headache intensity^1^						
	mean	2.4 +/− 0.5	2.2 +/− 0.6	2.3 +/− 0.7	2.1 +/− 0.7	ns
	median	2	2	2	2	ns
Pain free at						
	2 hours	10.7%	25.0%	12.5%	29.8%	p < 0.05^2^
	4 hours	35.7%	50.0%	25.0%	50.9%	ns
Recurrence at						
	24 hours	42.9%	37.5%	37.5%	26.3%	p < 0.05^3^
	48 hours	89.3%	91.7%	87.5%	66.7%	p < 0.01^4^

^1^Headache intensity graded as 0 = none; 1 = mild; 2 = moderate; 3 = severe.

^2^p < 0.05 for comparison between frovatriptan and rizatriptan.

^3^only for comparison between frovatriptan and rizatriptan.

^4^post-hoc analysis: p = 0.025 for frovatriptan versus rizatriptan; p = 0.019 for frovatriptan versus zolmitriptan; p = 0.232 for frovatriptan versus almotriptan.

Data are shown as mean (+/− SD), or frequency in %. Statistical comparison by ANOVA or Chi^2^-test (ns denotes not significant).

**Figure 1:**
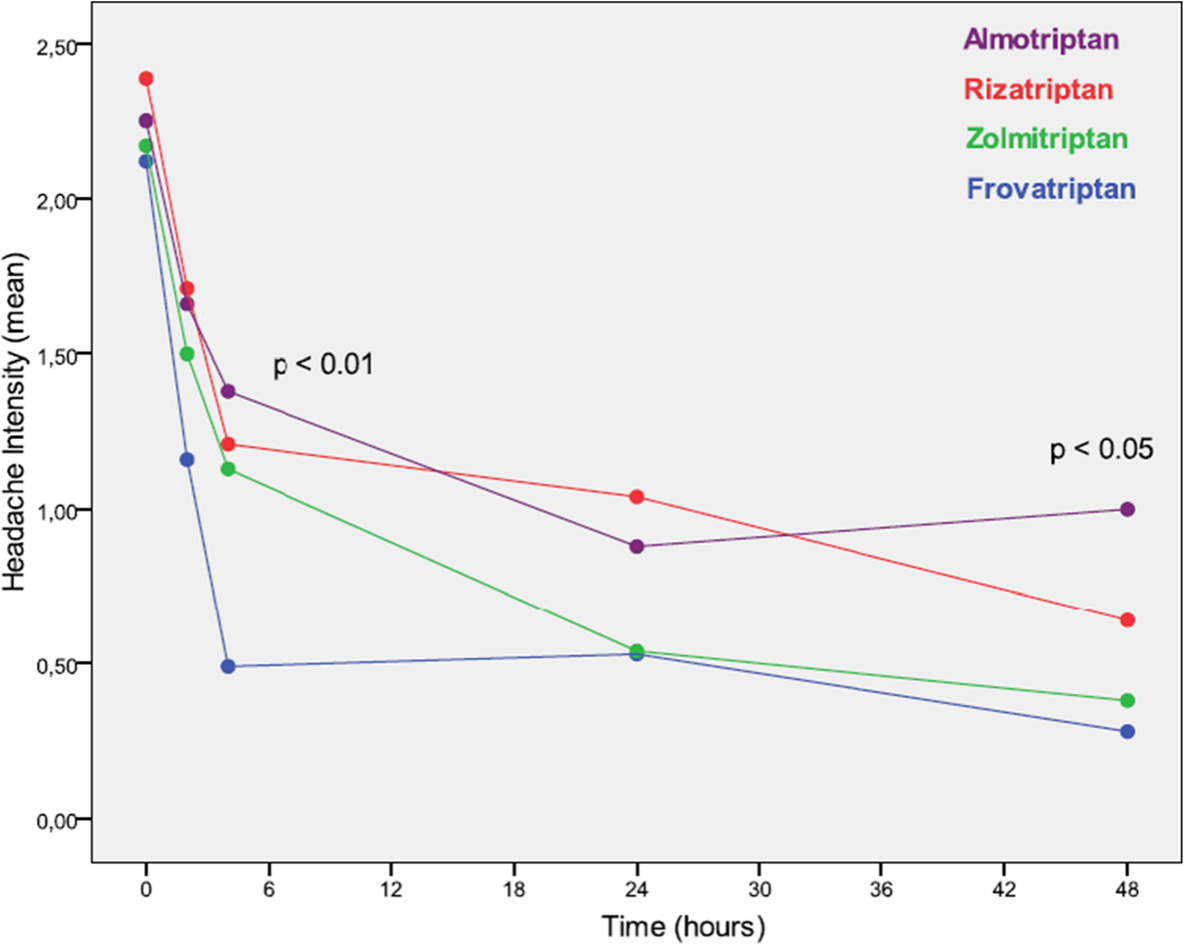
**Mean headache intensity** ^**1**^ **during attack treatment for all four different triptans. Statistical comparison by ANOVA, for post-hoc analysis see text.** ^1^Headache intensity graded as 0 = none; 1 = mild; 2 = moderate; 3 = severe.

In Figure 1, the mean headache intensity is presented for the period covering 48 hours after intake of the study drug. There was a significantly lower mean headache intensity for frovatriptan at 4 hours as compared to all other triptans and for frovatriptan and zolmitriptan at 48 hours as compared to rizatriptan and almotriptan (but not between frovatriptan and zolmitriptan). We also pooled the data from all comparative triptans. The mean headache intensity after 2 hours was 1.2 +/− 1.0 for frovatriptan and 1.6 +/− 1.0 for the other triptans (p < 0.05). After 4 hours, the mean headache intensity was 0.5 +/− 0.6 for frovatriptan and 1.2 +/− 1.1 for the other triptans (p < 0.001).

When analyzing the adverse events, there were no significant differences at all between the study drugs. The number and types of adverse events were quite similar to those seen in the migraine attacks without aura.

## Discussion

Our data show that frovatriptan results in a significantly lower relapse rate even when taken in acute migraine attacks with aura as compared rizatriptan, zolmitriptan, and almotriptan (the latter one not at 48 hours). This is in concordance with a previous analysis of all migraine attacks studied in a larger trial program [13]. Furthermore, this analysis confirms that triptans taken during the headache phase are in general efficacious and well tolerated in migraine with aura. This is of major importance since many patients experience migraine attacks both with and without aura; these patients do not have to change their way of acute attack treatment (e.g., the choice of a triptan) with respect to the aura.

The low recurrence rate of frovatriptan even in migraine with aura is also of interest for many patients as surveys on patients’ needs have shown [6,8]. In previous trials, frovatriptan was able to decrease the overall duration of migraine attacks significantly [19], and it is more efficacious when taken during the mild, beginning phase of a migraine attack versus taken during the later severe phase [20]. The short duration of attacks and the low recurrence rate result in a significantly lower mean headache intensity in this study after 24 hours and 48 hours. The significantly lower relapse rate of frovatriptan can be explained by its pharmacological properties [21]. When comparing all triptan trials, the elimination half-life time is inversely correlated with the relapse rate (r = −1.0; p = 0.0016). Frovatriptan has by far the longest half-life time (26 hours), whereas all the other oral triptans have a half-life time between 2 and 6 hours.

Some of our findings are surprising with respect to the literature, in particular the good efficacy of frovatriptan after 2 and 4 hours as compared to rizatriptan. The baseline characteristics of the migraine attacks and the MIDAS scores show that patients with mainly severe and long-lasting migraine attacks were enrolled into this study program. This might be the reason why frovatriptan, which is normally less efficacious in the first 2 hours after drug intake, was of particular efficacy in this study.

A limitation of this study is that it is a metaanalysis of different trials which were not designed to study the efficacy in migraine with aura as their primary endpoint. However, this metaanalysis is justified, since these trials all had a nearly identical design and since the two hour pain free rates in general were not significantly different between these triptans [13], i.e. the 2 hour pain free rate was not significantly lower for frovatriptan than for rizatriptan, zolmitriptan, or almotriptan. Previous analyses had suggested that the two hour pain free rate for frovatriptan is lower than for other triptans [11]; however, this has been shown in trials with treatment at any time during the migraine attack. When reevaluating this aforementioned finding in migraine attacks treated early, there was no significant difference between frovatriptan and other triptans regarding 2 hour pain free rates [11,22]. Also in the trials analysed here, patients were advised to treat their migraine attacks early.

Another limitation is that we did not extend our comparison to the remaining triptans (sumatriptan, naratriptan, eletriptan) or to other acute migraine drugs such as NSAIDs or ergotamine derivatives. Thus, the final position of frovatriptan within all acute migraine drugs with respect to efficacy in migraine with aura cannot be determined by this study.

Finally, the number of patients/attacks with aura was quite different between the different treatment groups. In particular, the comparison to almotriptan with only 8 attacks included in this analysis is problematic due to statistical reasons (large confidence intervals etc.).

## Conclusion

In summary, frovatriptan provides an efficacious treatment for migraine attacks with aura when taken during the headache phase with respect to acute efficacy and to relapse.
